# Mirror Symmetric Bimanual Movement Priming Can Increase Corticomotor Excitability and Enhance Motor Learning

**DOI:** 10.1371/journal.pone.0033882

**Published:** 2012-03-22

**Authors:** Winston D. Byblow, Cathy M. Stinear, Marie-Claire Smith, Lotte Bjerre, Brian K. Flaskager, Alana B. McCambridge

**Affiliations:** 1 Movement Neuroscience Laboratory, Department of Sport & Exercise Science, The University of Auckland, Auckland, New Zealand; 2 Center for Sensory-Motor Interaction (SMI), Department of Health Science and Technology, Aalborg University, Aalborg, Denmark; 3 Neurology Research Group, Department of Medicine, The University of Auckland, Auckland, New Zealand; 4 Centre for Brain Research, The University of Auckland, Auckland, New Zealand; University of Bologna, Italy

## Abstract

Repetitive mirror symmetric bilateral upper limb may be a suitable priming technique for upper limb rehabilitation after stroke. Here we demonstrate neurophysiological and behavioural after-effects in healthy participants after priming with 20 minutes of repetitive active-passive bimanual wrist flexion and extension in a mirror symmetric pattern with respect to the body midline (MIR) compared to an control priming condition with alternating flexion-extension (ALT). Transcranial magnetic stimulation (TMS) indicated that corticomotor excitability (CME) of the passive hemisphere remained elevated compared to baseline for at least 30 minutes after MIR but not ALT, evidenced by an increase in the size of motor evoked potentials in ECR and FCR. Short and long-latency intracortical inhibition (SICI, LICI), short afferent inhibition (SAI) and interhemispheric inhibition (IHI) were also examined using pairs of stimuli. LICI differed between patterns, with less LICI after MIR compared with ALT, and an effect of pattern on IHI, with reduced IHI in passive FCR 15 minutes after MIR compared with ALT and baseline. There was no effect of pattern on SAI or FCR H-reflex. Similarly, SICI remained unchanged after 20 minutes of MIR. We then had participants complete a timed manual dexterity motor learning task with the passive hand during, immediately after, and 24 hours after MIR or control priming. The rate of task completion was faster with MIR priming compared to control conditions. Finally, ECR and FCR MEPs were examined within a pre-movement facilitation paradigm of wrist extension before and after MIR. ECR, but not FCR, MEPs were consistently facilitated before and after MIR, demonstrating no degradation of selective muscle activation. In summary, mirror symmetric active-passive bimanual movement increases CME and can enhance motor learning without degradation of muscle selectivity. These findings rationalise the use of mirror symmetric bimanual movement as a priming modality in post-stroke upper limb rehabilitation.

## Introduction

Repetitive transcranial magnetic stimulation (rTMS) offers promise for increasing or decreasing M1 excitability to promote recovery of motor function after stroke [1]–[9], but a practical limitation is that it requires expensive equipment, a medical environment and is contraindicated for people with a history of seizure, metal implants, cardiac pacemaker, or who are taking certain common medications [10], [11]. Compared with rTMS, transcranial direct current stimulation (tDCS) has fewer contraindications but still requires the use of medically certified electrical equipment and application by a skilled operator [12]. Motor point stimulation [13], [14] and combined peripheral nerve and TMS can enhance or suppress M1 excitability through presumed spike-timing dependent mechanisms [15]–[17] but also require expensive equipment, skilled operators, or lengthy treatment periods and also has potential contraindications. The present study explores an alternative method for increasing M1 excitability by using patterned repetitive movement, without brain or nerve stimulation per se [18], [19].

It is well known that mirror symmetric bimanual movements, with homologous muscles activated simultaneously, are more stable than any other pattern [20]–[22]. Enhanced M1 excitability and presumed GABAergic M1 disinhibition have been noted during production of mirror symmetric active-passive bimanual movement [23], [24] and may facilitate upper limb recovery after stroke by acting as a neurophysiological priming mechanism [18], [19]. Until now, there has been no direct examination of M1 excitability and inhibition immediately after repetitive active-passive bimanual movement and no examination of the immediate behavioural consequences of active-passive movement priming.

To address these issues we first examined corticomotor excitability (CME), M1 intracortical and interhemispheric inhibition and H-reflex excitability, in healthy participants before and after 20 minutes (1200 cycles) of active-passive movement made in either a mirror symmetric (MIR) pattern, or an alternating (ALT) pattern. We hypothesised that MIR but not ALT movements would facilitate corticomotor excitability within forearm flexor and extensor representations of the passive left M1. We also predicted that any difference in CME noted between patterns would be accompanied by differences in intracortical inhibition. To examine this we obtained measures of short afferent inhibition (SAI), long-latency intracortical inhibition (LICI), interhemispheric inhibition (IHI) across two experiments, and examined H-reflex excitability in a third experiment. In a separate study we examined the behavioural consequences of active-passive movement priming and hypothesised that MIR priming would facilitate motor learning. Finally we examined whether increases in CME obtained after MIR would be associated with persistent reductions in short-latency intracortical inhibition that could potentially interfere with selective voluntary muscle activation.

## Materials and Methods

### Participants and Ethics Statement

Right-handed participants completed 1 or more experiments. Sample size, age, gender and handedness are provided in Table 1. Handedness was assessed using the Edinburgh Handedness Inventory [25], with a score of >+25 required for inclusion. The participants were neurologically healthy and with no upper limb injuries. The University of Auckland Human Participants Ethics Committee approved the protocol and participants gave written informed consent.

**Table 1:** Participant details, design, and summary of main results for each experiment.

	Exp 1	Exp 2	Exp 3	Exp 4			Exp 5
N	13	13	6	33 (3×11)			12
				MIR	ALT	NONE	
**Age (y)** **mean, range**	26.5,22–45	22.2,20–26	24.4,20–32	25.6,20–42	22.5,18–29	24.8,20–40	28.9,20–39
**Gender (M/F)**	6/7	6/7	2/4	4/7	5/6	2/9	6/6
**EH Score** **mean, range**	82.0,50–100	81.7,58–100	80.1,70–100	76.1,42–100	80.0,60–100	79.3,64–100	79.4,44–100
**Design**	1 group2 session	1 group2 session	1 group2 session	3 group2 session			1 group1 session
**Active-passive movement**	MIR, ALT	MIR, ALT	MIR, ALT	MIR, ALT, NONE			MIR
**Neurophysiology**	CME, LICI, SAI	IHI	H-reflexes	-			CME, SICI
**Function**	-	-	-	Motor learning			Selective facilitation
**MIR effects**	↑ CME↓ LICI*←→ SAI	↓ IHI	←→ H amp	↑ rate of learning			↑ CME←→ SICI←→ PMF

Exp = Experiment; N = number of participants; EH = Edinburgh Handedness (−100 = left-handed; +100 = right-handed) MIR = mirror-symmetric; ALT = alternating; CME = corticomotor excitability of the passive M1; SAI = short afferent inhibition; LICI = long interval intracortical inhibition; IHI = Interhemispheric inhibition; SICI = short interval intracortical inhibition; H amp = H-reflex amplitude; PMF = pre-movement facilitation; ↑ Increase; ↓ Decrease; ←→ No change. All MIR effects are relative to baseline, except * = relative to ALT.

### Design and basic protocol

Table 1 summarises the design of each experiment (Exp). Exp 1–3 required two sessions, one for each active-passive movement pattern, but were otherwise identical. Sessions were separated by at least three days and session order was randomised between participants. In Exp 1, CME, SAI and LICI were investigated before and after active-passive movement using single and paired-pulse TMS targeting right extensor carpi radialis (ECR) and flexor carpi radialis (FCR) representations in left M1. In Exp 2, IHI from right to left M1 was examined before and after active-passive movement using dual-coil TMS targeting both left and right FCR. In Exp 3, right FCR H-reflexes were examined before and after active-passive movement. Data were collected at four times: before movement (Pre / Baseline), immediately (Post_0_), 15 and 30 minutes after movement (Post_15_ and Post_30_).

In Exp 4, behavioural effects of active-passive movement were measured using a timed grooved pegboard test (GPT, Lafayette instrument company, Lafayette, IN USA). Participants were randomised into 3 groups, performing either MIR, ALT or no (NONE) movement priming, and the nondominant (left) hand was passive during MIR and ALT. The GPT is a timed, manipulative dexterity test consisting of placing pegs into 25 holes. Pegs have a square edge or key on one side such that the peg must be manipulated in the hand precisely to align with each hole (with key slots of varying orientation). The participant was seated at a table with the chair adjusted to a height so that the participant's forearm was able to rest on the table with the elbow in 90° of flexion. The nondominant left hand was used to place the pegs. On each attempt participants were instructed to complete the pegboard test as quickly as possible. An initial measure was taken as baseline, followed by four attempts during, immediately after, and 24 h after active-passive movement priming or no movement priming. The time between attempts was 5 min.

In Exp 5, participants completed one session of MIR active-passive movement. CME and short latency intracortical inhibition (SICI) were investigated before and after MIR using single and paired-pulse TMS, targeting right ECR and FCR representations in left M1. Pre-movement facilitation (PMF) was examined before and after MIR using single pulse TMS delivered within the reaction time (RT) interval of right wrist extension. Data were collected at 3 time points: baseline, immediately after APBT (Post_0_) and 30 minutes after APBT (Post_30_).

### Active-passive movement

**Figure 1:**
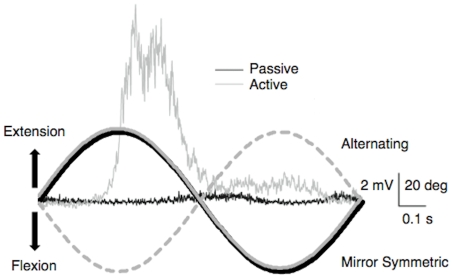
The active-passive movement protocol. Average left (active) and right (passive) FCR EMG traces from a single participant (1 s of data, average of 60 traces). For clarity EMG is shown from a mirror symmetric condition only. The marked difference in EMG activity can be seen between the active FCR (grey trace) and passive FCR (black trace) during the movement. A schematic of passive (black trace) and active wrist angle from mirror symmetric and alternating session of Exp 1 is shown.

For Exp 1–3 participants sat in a custom apparatus [23] that maintained the shoulders in slight abduction (10–20°), elbows flexed at 90–110°, and forearms supported in a neutral position. Each hand was secured in a support that allowed up to 100° flexion/extension movements of each wrist joint. The apparatus was comprised of two manipulanda, coupled together via a torque-motor system, so that active flexion-extension of the left wrist would passively flex and extend the right wrist in a synchronized mirror symmetric or parallel movement pattern. Briefly, the “passive” manipulandum housed a brushless AC torque motor (Baldor, Fort Smith, AR, USA) driven by a Baldor D3S motor drive, a motion control board (Delta Tau Data, Northridge, CA, USA) and a PC running custom software (LabVIEW, National Instruments). The “active” manipulandum was without a motor. A voltage signal from a potentiometer mounted to the rotating axis beneath the left wrist was converted in software and used as an input to the torque motor for precise mirror symmetric (MIR, passive flexion during active flexion) or alternating (ALT, passive extension during active flexion) movements of the passive hand with respect to the active. In each session participants produced 1200 cycles (60 cycles/min×20 min) of active-passive wrist flexion-extension (1 cycle = 80°, 40° either side of neutral). The target amplitude was indicated with padded stops on the active manipulandum. Participants reached peak flexion with the active hand in time with a 1 Hz metronome, and reached peak extension between each beat. At the same time the right hand was driven through wrist flexion-extension by the torque motor to match the frequency and amplitude of the active hand (temporal resolution <1 ms, spatial resolution <1°) in either a MIR or ALT pattern. A screen was placed over the right manipulandum to obscure vision of the right arm, and to assist relaxation of the right arm. During active-passive movement, 50 epochs of raw EMG (1 s duration, 500 ms pre-trigger) were collected at minute 7 and 14, triggered at the midway point between peak flexion and extension. Example hand displacement and EMG traces of active left FCR and passive right FCR are shown in Figure 1.

In Exp 4, subjects assigned to MIR or ALT groups again used the torque motor manipulandum described above, but this time with the left side passive. This was done so that learning could be assessed using the nondominant left hand. Participants assigned to a no movement (NONE) group did not perform any active-passive movement. In Exp 5 participants used a table-top mechanical device designed for producing MIR active-passive wrist-flexion extension without the need for motorised drive. Each hand was secured in a hand-piece which allowed flexion and extension movements of the wrist up to 100°. The device mechanically couples the hands to move in MIR, driven by voluntary movement on one side while the other remains passive. Participants were instructed to actively flex and extend their left wrist while keeping the right hand passive in time with a 1 Hz metronome (full flexion on the beat, full extension between beats). As in other experiments, participants completed 1200 cycles of flexion/extension in 20 minutes and EMG data were collected at minute 7 and minute 14.

### Electromyography (EMG)

Surface EMG of left and right ECR and FCR was recorded using 10 mm diameter Ag/AgCl electrodes (Ambu, Ballerup, Denmark) placed 2 cm apart over the muscle bellies, following standard skin preparation techniques. A common ground surface electrode (3M Health Care, Canada) was placed on each elbow. In Exp 1–3 EMG signals were amplified (Grass P511AC, Grass Instrument Division, West Warwick, RI), band-pass filtered (3 Hz–1 kHz), using custom software (LabVIEW). In Exp 4, EMG signals were amplified (CED 1902; Cambridge Electronic Design, Cambridge, United Kingdom), band-pass filtered (20–1000 Hz), and sampled at 2 kHz (CED 1401) and stored for offline analysis (Signal V4.09) .

### Transcranial magnetic stimulation (TMS)

In Exp 1 and 5, single or paired-pulse TMS of left M1 was delivered using two Magstim Model 200 stimulators connected to a BiStim unit (Magstim Company, Dyfed, UK). A figure of eight coil (70 mm wing diameter) was held tangentially to the scalp with the handle pointing backwards and laterally at an angle of approximately 45° in the sagittal plane. The induced current flow was in a posterior to anterior direction along the motor strip. The coil was positioned at the optimal site for producing maximal responses in the resting ECR and FCR muscles and this spot was marked on the scalp to ensure consistent coil placement throughout the experiment. In Exp 2, two 55 mm diameter figure of eight coils were positioned to induce posterior to anterior current in left and right M1, at the optimal sites for producing maximal responses in the resting FCR muscles.

### Data Collection and Dependent Measures

During MEP collection the participant sat comfortably with their hands resting in their lap, outside of the active-passive apparatus.

#### Resting Motor Threshold (RMT)

Resting motor threshold (RMT) was defined as the minimum intensity for eliciting MEPs of at least 50 µV peak-peak amplitude in four of eight trials in the relaxed ECR muscle [26].

#### MEP area

Due to the polyphasic nature of forearm muscle MEPs, MEP area (mV×ms) was used as the primary dependent measure, calculated over a 20 ms window from MEP onset determined individually for each muscle and participant (Stinear and Byblow, 2004b). MEP areas obtained at post time points were normalised to baseline. TMS intensity was set to produce non-conditioned (NC) MEPs that were 50% of the maximal MEP obtainable in ECR, to ensure MEP amplitude was on the linear part of the stimulus-response curve [27]. The root mean square (rmsEMG, mV) of the pre-trigger EMG was determined over the period 105–200 ms prior to the test stimulus to avoid contamination due to conditioning stimulus artefact. Traces with pre-trigger rmsEMG activity >10 µV in either muscle were discarded.

#### Short Afferent Inhibition (SAI)

Cutaneous stimulation of the right index finger (D2) was applied (Digitimer DS7, 0.1 ms duration, 150 V) through a pair of ring electrodes with the cathode at the proximal part of D2 and the anode 2 cm distal. The intensity was set to 3× perceptual threshold. To examine SAI, the peripheral stimulus was delivered 40 ms prior to TMS of left M1 yield conditioned (C) MEPs (Helmich et al., 2005). Twenty NC and C MEPs were collected in random order at each time point.

### Long-latency intracortical inhibition (LICI)

**Figure 2:**
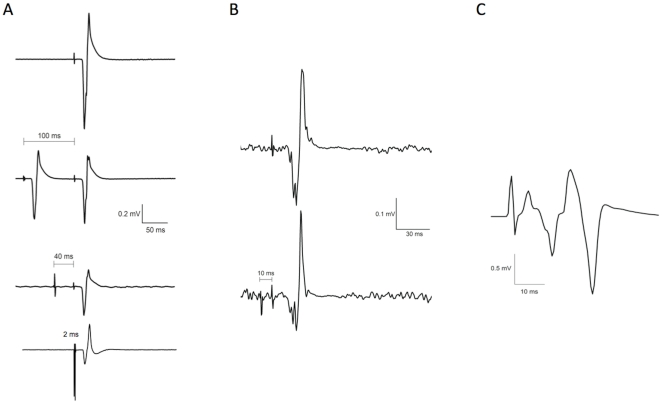
MEPs and H-reflexes (A–C) were obtained from passive FCR at baseline and 0, 15 and 30 minutes after 20 minutes of active-passive movement made in either a mirror symmetric or alternating pattern. The only difference between sessions was the phase relation of the active hand with respect to the passive hand. **A.** Intracortical Inhibition Protocols (Top to Bottom): Single-pulse TMS of left M1 elicits a non-conditioned MEP. The test stimulus intensity is set to produce an MEP roughly half of the maximum amplitude obtainable in that muscle. Long interval intracortical inhibition (LICI, Exp 1) was examined with two supra-threshold stimuli applied 100 ms apart. Short interval afferent inhibition (SAI, Exp 1) was examined by applying cutaneous stimulation through ring electrode on the right index finger 40 ms prior to TMS at the test intensity. Short interval intracortical inhibition (SICI, Exp 5) was examined by applying a subthreshold stimulus 2 ms before the test stimulus. Suppression of the test MEP is evident during LICI, SAI and SICI protocols. Each trace is the average of twenty sweeps. **B.** Interhemispheric Inhibition. Top: Single-pulse TMS of left M1 elicits a test MEP in pre-activated right FCR. Bottom: To examine interhemispheric inhibition (IHI, Exp 2), TMS of right M1 is applied 10 ms before TMS of left M1. Suppression of the test MEP is evident. Each trace is the average of twenty sweeps. **C.** H-Reflex. H-reflexes were obtained in the resting right FCR (Exp 3). The stimulus artefact is followed closely by an M-wave that is approximately 10% of the maximum M-wave amplitude (not shown), followed by the H-wave. The trace is an average of thirty sweeps.

Single-coil paired-pulse TMS was delivered with the intensity of both stimulators set as described above and an interstimulus interval (ISI) of 100 ms [28]. Twenty NC and C MEPs were collected in random order at each time point. Example EMG traces showing NC MEPs, LICI and SAI are shown in Figure 2a.

#### Interhemispheric Inhibition (IHI)

During the protocol the participants sat comfortably with their right forearm supinated on their lap and the wrist flexed to support a 1 kg weight just above their lap outside of the active-passive movement apparatus. The right FCR was pre-activated to ensure suitably large MEPs were obtained. Dual-coil paired-pulse TMS was delivered with an ISI of 10 ms. TMS intensity of left M1 was set to produce a MEP in right FCR that was 50% of maximum. A conditioning pulse was applied to the right M1 with TMS intensity set to produce approximately 50% of the maximum inhibition of the NC MEP at baseline [29]. Minimum and maximum pre-trigger rmsEMG levels in right FCR were set to ±5% MVC to ensure contraction level remained consistent and traces were rejected online when EMG levels were out of range. Thirty MEPs were collected at each time point, randomised between C and NC. Example EMG traces illustrating IHI are shown in Fig. 2b.

### Median Nerve Stimulation: M-Wave and H-Reflex

During data collection the participants sat comfortably with their hands supine and resting in their lap. M-waves and H-reflexes were recorded from the right FCR by stimulating the median nerve (Digitimer DS7 stimulator, 1 ms square wave pulse, stimulation rate 0.3 Hz). The cathode was placed on the skin over the nerve on the medial surface of the upper arm where the nerve courses superficially along the border of the adjacent biceps and triceps brachii. The anode was placed over the ipsilateral acromion process. The amplitude of the maximum M-wave (M_max_) was determined. Stimulator intensity was then adjusted to produce an M-wave approximately 10% M_max_, where it was confirmed that the H-reflex was on the ascending part of the S-R curve. At each time point the M-wave amplitude was monitored, and stimulus intensity adjusted if necessary, to obtain 10% M_max_. Thirty H-reflexes were collected at each time point. Example EMG traces illustrating M wave and H reflexes are shown in Fig. 2c.

### Motor Learning Task

The GPT completion times (s), and the slope of times within each block, were used as measures of learning for each participant. The time taken at baseline was used to normalise all other times to control for individual differences due to age, gender and hand size. The rate of learning was quantified by computing the slope of the normalised times of the four attempts within each block using linear estimation. A negative slope indicates the average rate of improvement over the block, with steeper slopes indicative of a faster rate of learning.

### Corticomotor Excitability and Short-latency Intracortical Inhibition (SICI)

Active motor threshold (AMT) of the left M1 was determined as the minimum stimulus intensity required to elicit a MEP of >50 µV peak-to-peak amplitude in at least 4 out of 8 consecutive trials in the pre-activated right ECR muscle held in 10° wrist extension. TMS intensity was set to produce ECR NC MEPs of 50% of maximum amplitude, as described above. Conditioning stimulus intensity was set to AMT of ECR, and preceded the test stimulus by 2 ms [30]. Pre-trigger EMG was collected for 100 ms and traces with pre-trigger rmsEMG>10 µV were discarded. Twelve NC and 12 C stimuli were delivered at rest and NC and C MEP areas were calculated as described above.

### Pre-movement facilitation / Reaction Time task

Participants were seated with their right elbow flexed to 90 degrees and forearm resting in pronation on a table placed at their right side. Right wrist extension was performed as a simple reaction time (RT) task in response to an auditory cue. Participants initially completed 20 trials without TMS in order to establish baseline RT. There was an interval of 6 seconds between each auditory cue with a random variation of +/−30%. RT was defined as the time between the auditory cue and the onset of the EMG response. The ECR EMG from 20 trials was rectified, averaged and used to determine baseline RT using a semi-automatic detection threshold of 5 standard deviations from background rmsEMG. To investigate pre-movement facilitation (PMF), single-pulse TMS was delivered at a time corresponding to 25% (early) and 75% (late) of the individual's baseline RT in a randomised order (48 trials in total) following an established paradigm [31]. An additional twelve trials without TMS were also randomly interspersed as a check on RT.

### Statistical analysis

SAI, LICI, IHI, and SICI were expressed as a percentage using a formula of % Inhibition, %INH = 100−(C/NC×100), where C and NC correspond to conditioned and non-conditioned MEP area respectively. H-reflex amplitude was expressed as a percentage of M-Max. Baseline values were subtracted from values obtained at each subsequent time point to obtain measures of ΔSAI, ΔLICI, ΔIHI and ΔH-reflex.

For Exp 1, a three-way repeated measures analysis of variance (rmANOVA) with factors Muscle (ECR, FCR), Pattern (MIR, ALT), and Time (Post_0_, Post_15_, Post_30_) was used to examine NC MEP area, ΔSAI and ΔLICI. A similar rmANOVA was performed for pre-trigger rmsEMG, except with four levels of Time to include baseline (Pre). Paired-t-tests were used to explore interactions and main effects with more than two levels. One sample t-tests were used to determine if post values were significantly different from baseline.

For Exp 2, a two-way rmANOVA with factors Pattern and Time was used to assess changes in ΔIHI. Non-conditioned MEP area as a percentage of baseline was assessed using a two-way rmANOVA with factors Pattern and Time. Pre-trigger rmsEMG was assessed with a three-way rmANOVA with factors Pattern, Conditioning (NC, C) and Time (4 levels). Paired t-tests were used to explore main effects and interactions.

For Exp 3, a two-way rmANOVA with factors Pattern and Time was used to assess ΔH-reflexes. Pre-trigger EMG was assumed quiescent since all testing was done at rest. M-wave amplitude was expressed as %M_MAX_ and analysed using a two-way rmANOVA with factors Pattern and Time (4 levels).

For Exp 4, independent sample t-tests of the pegboard completion times at baseline were used to determine if the Groups were balanced. As an index of overall learning a 3 Group×12 Trial mixed ANOVA was conducted on normalised times to determine if there was an effect of Trial. Differences between Groups were then explored within a Block to differentiate between trials that were undertaken during priming, afterward, and at retention. This was done by fitting a linear equation to normalised times within each block and then estimating the slope of the line. First, a mixed ANOVA of normalised slope (% baseline) indicated there were no differences between the two control conditions (ALT and NONE), so these were combined into a single control (CON) group. Then a 2 Group (MIR, CON)×3 Block (During, After, Retention) mixed ANOVA of normalised slope was performed.

For Exp 5, normalised NC MEP area (% baseline) was examined using a two-way rmANOVA with factors Muscle (ECR, FCR) and Time (Post_0_, Post_30_). A similar rmANOVA was used to examine SICI across 3 time points (Pre, Post_0_, Post_30_). A three-way rmANOVA was used to examine PMF with factors Muscle (ECR, FCR), Time (Pre, Post_0_, Post_30_), and Phase (Early, Late). Reaction time (RT) without TMS was examined using a one-way rmANOVA with main factor Time (Pre, Post_0_, Post_30_). Pre-trigger rmsEMG was examined with three-way rmANOVAs with factors Muscle (ECR, FCR), Time (Pre, Post_0_, Post_30_) and Conditioning (NC, C) for SICI trials, and with factor of Phase (Early, Late) instead of Conditioning for PMF trials.

During active-passive movement, for Exp 1 and 5, ECR and FCR rmsEMG was collected from 120 1-s epochs collected at minutes 7 and 14. Root mean square EMG was averaged across all epochs and analysed using a three-way rmANOVA, with factors Muscle, Pattern (Exp 1 only) and Arm (active, passive). For Exp 2 and 3, FCR rmsEMG was collected from 120 1-s epochs at minute 7 and 14, averaged across epochs, and analysed using a two-way rmANOVA, with factors Pattern and Arm. For Exp 4 EMG was collected in the passive ECR and FCR as a manipulation check only, and only for participants that performed active-passive movement (MIR and ALT Groups).

Statistical results were deemed significant if P<0.05. Greenhouse Geisser corrections were undertaken when sphericity was violated. For measures of %INH, planned comparisons were undertaken with one-sample t-tests of %INH against baseline. Paired t-tests or independent sample t-tests (Exp 4) were used to explore interactions and main effects with more than two levels. Post-hoc tests were corrected for multiple comparisons when required [32]. Values are presented in text as mean ± standard error (SE).

## Results

### Exp 1 – Effect of active-passive movement on corticomotor excitability and intracortical inhibition

#### Stimulation intensity and rest motor threshold (RMT)

There were no differences between sessions in test TMS intensity or RMT for left M1 at baseline (Table 2). After active-passive movement there was a trend for RMT to decrease with Time (F_2,24_ = 3.36, *P* = 0.056) but with very little change relative to baseline (100%) (Post_0_: 101.6±0.8% ; Post_15_: 100.4±1.0%; Post_30_: 99.4±1.2%). There was no effect of Pattern or Pattern×Time interaction (both *P*>0.3), indicating that RMT was stable within and between sessions.

**Table 2:** Group mean (± SE) baseline measures from each session (Exp 1–3).

	BaselineMeasures	MirrorSession	AlternatingSession	dF	*P*
**Exp 1**	RMT (% MSO)	51.4±3.5	52.3±3.2	12	0.63
	SI (% MSO)	70.0±2.9	70.9±2.7	12	0.90
	ECR NC MEP Area (mV⋅ms)	2.7±0.7	3.5±1.0	12	0.20
	FCR NC MEP Area (mV⋅ms)	2.6±0.5	4.0±1.5	12	0.22
	ECR SAI (%INH)	64.2±17.1	53.5±11.3	7	0.42
	FCR SAI (%INH)	35.5±9.7	33.2±5.5	7	0.85
	ECR LICI (%INH)	40.5±15.8	47.6±15.2	7	0.56
	FCR LICI (%INH)	54.8±12.4	56.7±11.7	7	0.90
**Exp 2**	FCR IHI (%INH)	20.1±4.1	21.5±4.2	12	0.70
	FCR NC MEP Area (mV⋅ms)	5.2±0.6	5.5±0.5	12	0.45
	FCR rmsEMG (µV)	31.7±2.4	28.5±2.4	12	0.13
**Exp 3**	M_MAX_ (mV)	7.4±1.5	7.3±1.14	5	0.90
	M-wave (%M_MAX_)	12.4±1.3	12.8±1.6	5	0.91
	H-reflex (mV)	1.76±0.3	1.56±0.4	5	0.71

Abbreviations as in Table 1. RMT = Resting Motor Threshold; MSO = Maximum Stimulator Output; SI = Stimulation Intensity; ECR = Extensor carpi radialis; NC MEP = Non-conditioned motor evoked potential; FCR = Flexor carpi radialis; %INH = Percent Inhibition (Note: larger values indicate greater inhibition); rms = root mean square; M_MAX_ = Maximum M-wave.

#### Non-conditioned ECR and FCR MEP area

**Figure 3:**
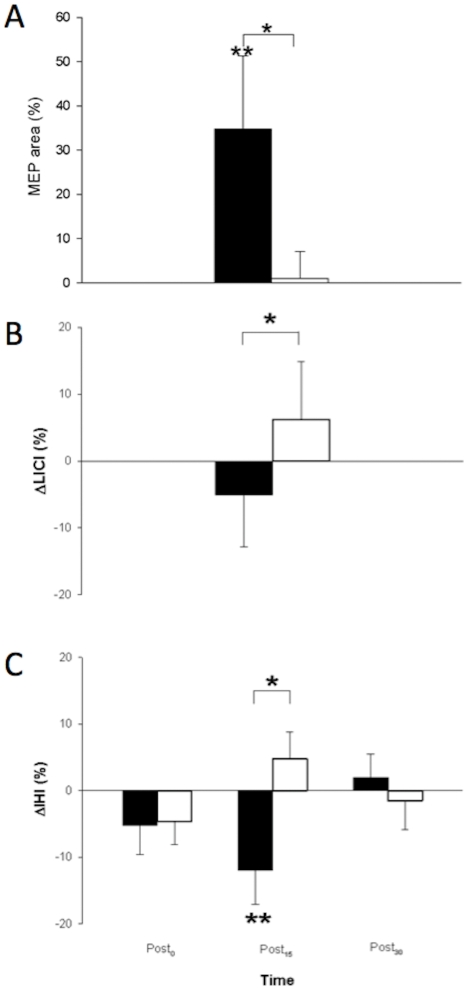
Neurophysiological effects after active-passive movement priming. Black bars are group averages from the mirror symmetric (MIR) session; white bars are group averages from the alternating (ALT) session. **A.** Corticomotor excitability increased after MIR but not ALT. There was a main effect of Pattern for non-conditioned ECR and FCR MEP area (Exp 1). Bars represent average ECR and FCR MEP area from 13 participants collapsed across all three post time points expressed as a percentage of baseline (100%). One-sample t-tests indicated significant MEP facilitation in both muscles after mirror symmetric but not parallel movement. **B.** Long interval intracortical inhibition (LICI) was modulated by Pattern (Exp 1). There was a main effect of Pattern for ΔLICI. Bars represent average change in LICI from ECR and FCR MEPs of 8 participants at each Post time point relative to baseline (0%). Relative to baseline, there was a non-significant trend for reduced LICI after the mirror session (*P*<0.1) and a trend for increased LICI in the alternating session (*P*<0.06). **C.** Interhemispheric inhibition (IHI) was modulated by Pattern and Time reduced after the MIR, but not PAR, session (Exp 2) indicated by a Pattern×Time interaction for ΔIHI. Bars represent average change in IHI from FCR MEPs of 13 participants at each Post time point relative to baseline (0%). At Post_15_, ΔIHI was less in the MIR session compared to the ALT session, but did not differ at other time points. One-sample t-tests indicated a significant reduction of IHI at Post_15_ relative to baseline after MIR only.

At baseline there were no differences in NC MEP area between sessions (Table 2). After active-passive movement, there was a significant main effect of Pattern (F_1,12_ = 4.74, *P*<0.05) with larger non-conditioned MEP areas after MIR than ALT (Figure 3a). There were no other main effects or interactions (all *P*>0.1). One-sample t-tests indicated significant facilitation of MEPs after MIR (t_77_ = 4.49, *P*<0.001, corrected, +34.75±7.73%) but no change after ALT (t_77_<1, +0.97±3.79%). There was a lasting increase in CME in the passive hemisphere for both ECR and FCR muscle representations for at least 30 min after MIR but not ALT active-passive movement.

#### Short-latency afferent inhibition

Eight of 13 participants demonstrated at least 10% MEP suppression in ECR or FCR during the SAI protocol, and their data were included in further analyses. At baseline SAI ranged from 53–64 %INH in ECR and 33–35 %INH in FCR, confirming the protocol for these participants. SAI at baseline did not differ between sessions (Table 2). After active-passive movement, there were no significant main effects or interactions on ΔSAI (all *P*>0.1).

#### Long-interval intracortical inhibition

The paired-pulse protocol produced at least 10% suppression of MEPs in eight of thirteen participants and their data were included in further analyses. At baseline LICI ranged from 40–48 %INH in ECR and 54–57 %INH in FCR, confirming the protocol for these participants. LICI at baseline did not differ between sessions (Table 2). After active-passive movement, analysis of ΔLICI indicated a significant main effect of Pattern (F_1,7_ = 6.32, *P* = 0.040) with less LICI in the passive M1 after the MIR session compared to the ALT session (Figure 3b). There were no other main effects or interactions (all *P*>0.4). Data were pooled across Muscle and Time to explore the effect of Pattern. One-sample t-tests indicated that after ALT there was a trend toward increased LICI (t_47_ = 1.88, *P* = 0.067, uncorrected, +1.88±3.45%) but after MIR, LICI was not different from baseline (t_47_ = −1.60, *P* = 0.117, uncorrected, −1.60±3.18%).

#### Pre-trigger RMS EMG

Both ECR and FCR were quiescent during TMS with pre-trigger rmsEMG levels below 10 µV (ECR: 8.0±1.0 µV, FCR 4.0±1.0 µV). There was a significant main effect of Muscle (F_1,12_ = 599.9, *P*<0.0001), but no other effects or interactions (all F<1). The difference between muscles likely reflected a difference between the EMG amplifiers dedicated to recording of each muscle.

#### Active-passive movement

After baseline measurements, all participants completed 1200 cycles of active-passive movement in 20 minutes. All participants found the movements easy to perform and indicated no difference in difficulty between MIR and ALT sessions. Participants were successful at maintaining relative muscle quiescence in the passive arm. Mean rmsEMG of the active ECR and FCR was 64±1 and 76±2 µV respectively. The mean passive rmsEMG was 14±1 µV. During active-passive movement there were no significant main effects or interactions for background rmsEMG of the passive ECR and FCR (all *P*>0.06).

### Exp 2 - Effect of active passive movement on interhemispheric inhibition

In Exp 1, MIR but not ALT movements induced persistent increases in corticomotor excitability, perhaps in part due to differential modulation of GABA_B_ receptor activity (as evident by ΔLICI) between patterns. To further explore the mechanisms of this pattern-dependent change in CME, we investigated IHI before and after MIR or ALT active-passive movement in separate sessions.

#### Non-conditioned FCR MEP area

At baseline, NC MEP area in voluntarily activated FCR did not differ between sessions (Table 1). After active-passive movement there was a main effect of Time (F_2,24_ = 10.38, *P* = 0.002), but no effect of Pattern or interaction (all *P*>0.1). NC MEP area in pre-activated FCR was significantly suppressed from baseline at Post_0_ (83.1±3.4%, t_25_ = −4.99, *P*<0.001, corrected) but not at other times (95.8±4.9% at Post_15_, and 101.9±4.3% at Post_30_, both *P*>0.4).

#### Interhemispheric Inhibition

The left hemisphere test stimulus intensity was 59.5±3.0 %MSO and the right hemisphere conditioning stimulus intensity was 63.3±2.8 %MSO. Individual TS and CS intensities were kept identical between the two sessions. On average the IHI protocol produced approximately 20% suppression of FCR MEPs for all participants at baseline in both MIR and ALT sessions, confirming the protocol.

There was no difference in IHI at baseline between sessions (Table 2). Interhemispheric inhibition was reduced after MIR compared with ALT movement. Analysis of ΔIHI indicated a Pattern×Time interaction (F_2,24_ = 3.79, *P* = 0.040), without main effects of either (all *P*>0.1) (Figure 3c). Paired t-tests indicated that ΔIHI differed between MIR (−12.0±5.2%) and ALT (4.8±4.0%) at Post_15_ only but not at any other time point (Post_15_: t_12_ = −2.64, *P* = 0.022, corrected, all others *P*>0.1). One-sample t-tests indicated that IHI was reduced from baseline at Post_15_ only after MIR (t_12_ = 2.32, *P* = 0.039, corrected) and unchanged at any time point after ALT (*P*>0.2).

#### Pre-trigger rmsEMG

There were no differences between sessions at baseline for FCR pre-trigger rmsEMG levels (Table 2). As expected, after active-passive movement there were no main effects of interactions (Pattern×Time interaction: *P* = 0.068, all others *P*>0.1).

#### Active-passive movement

After baseline measurements, all participants completed 1200 cycles of active-passive movement in 20 minutes. All participants found the movements easy to perform and indicated no difference in difficulty between MIR and ALT sessions. The rmsEMG of the active left FCR (40±4 µV) was greater than the left passive FCR (16±2 µV) as revealed in the expected main effect of Arm (F_1,12_ = 42.20, *P*<0.0001), with no other main effects or interactions (all *P*>0.1).

### Exp 3 – Effect of active passive movement on H-reflex excitability

#### M-wave and H-reflex amplitude

FCR H-reflexes were reliably obtained in six participants. At baseline there were no differences between sessions for M_max_, M-wave amplitude, or H-reflex amplitude (Table 2). After active-passive movement H-reflexes tended to be suppressed slightly, but there were no main effects or interactions for ΔH-reflex (all *P*>0.25). Averaged across Time, ΔH-reflex after MIR was −4.41±3.65 (%M_MAX_) and after ALT, −3.35±4.35 (%M_MAX_). One-sample t-tests indicated H-reflexes were suppressed for a brief period relative to baseline after MIR (Post_0_ −3.9±1.0 %M_MAX_, t_5_ = −4.11, *P* = 0.009 corrected) but not at any other time point, nor at any time after ALT (all *P*>0.13). M-wave amplitude (%M_MAX_) was stable throughout the procedure with no effects or interactions of Pattern or Time (averaged across Time, MIR = 12.3±1.5%M_MAX_; ALT = 12.2±1.5%M_MAX_, all *P*>0.2).

#### Active-passive movement

After baseline measurements, all participants completed 1200 cycles of active-passive movement in 20 minutes. All participants found the movements easy to perform and indicated no difference in difficulty between MIR and ALT sessions. Again, there was a main effect of Arm (F_1,5_ = 129.68, *P* = 0.000), with no other main effects or interactions (all *P*>0.1). The rmsEMG was greater in the active FCR (37±2 µV) compared with the passive FCR (11±1 µV).

### Exp 4 – Effect of active-passive movement on motor skill acquisition

#### Motor Learning: Grooved Pegboard Test

**Figure 4:**
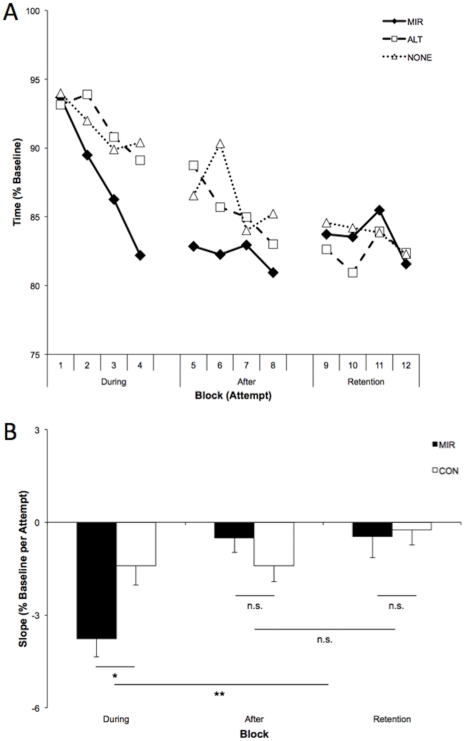
Functional effects of active-passive movement priming on motor learning (Exp 4). **A.** Normalized times taken to complete Grooved Pegboard test for MIR, ALT and NONE conditions. Each point represents the average of 11 participants. **B.** Rate of learning was quantified as the slope of normalized times obtained within each Block. ALT and NONE did not differ and were combined into a single control group (CON, N = 22). A Mixed ANOVA indicated a main effect of Block and a Group×Block interaction. Slopes were steeper in Block 1 than Block 2 and 3, which did not differ. MIR slope was steeper than CON for During Block only. * *P*<0.05; ** *P*<0.005.

The time to complete the grooved pegboard test at baseline did not differ between Groups (MIR = 73.1±2.8 s; ALT = 72.3±2.2 s; NONE = 72.0±2.5 s, all *P*>0.8). Normalised times and slopes are presented in Figure 4a & b. The analysis of normalised times yielded the expect main effect of Trial (*F*_11,330_ = 15.30, *P*<0.001) with strong linear and quadratic trends (*F*_1,30_ = 78.73, *P*<0.0001 and *F*_1,30_ = 14.59, *P* = 0.001 respectively). There was no Group×Pattern interaction (*F*_22,330_ = 1.47, *P* = 0.082), although a cubic trend in the interaction term was evident (*F*_2,30_ = 4.52, *P*<0.02, see Fig. 4a).

Analysis of normalised slope between the two control conditions ALT and NONE indicated no main effects or interaction (Group: *F*_1,20_<1, *P* = 0.97; Block *F*_2,40_ = 1.44, *P* = 0.25; Group×Block *F*_2,40_<1) so these groups were combined into a single control (CON) for comparison with MIR. The mixed ANOVA of normalised slope indicated a main effect of Block (*F*_2,62_ = 6.79, *P* = 0.002) with a linear relationship (*F*_1,31_ = 11.10, *P* = 0.02), and a Group×Block interaction (*F*_2,62_ = 3.49, *P* = 0.037) with a quadratic relationship (*F*_1,31_ = 4.65, *P* = 0.031). As can be seen in Figure 3b, the interaction arose because the negative slope indicative of learning was steeper During MIR priming than During CON priming (*t*_31_ = −2.42, *P* = 0.022), but with no differences between Groups in the subsequent Blocks (After: *P* = 0.261; Retention: *P* = 0.806).

#### Active-passive movement

After baseline measurements, participants in MIR and ALT Groups completed 1200 cycles of active-passive movement in 4×5 minute bouts interspersed with pegboard test attempts. The rmsEMG indicated muscles in the passive arm remained at rest (ECR: MIR 10±0.4 µV, ALT 12±0.2 µV; FCR: MIR 10±0.7 µV; ALT 13±0.4 µV).

### Exp 5 – Effect of MIR on SICI and pre-movement facilitation

#### Non-conditioned ECR and FCR MEP area

**Figure 5:**
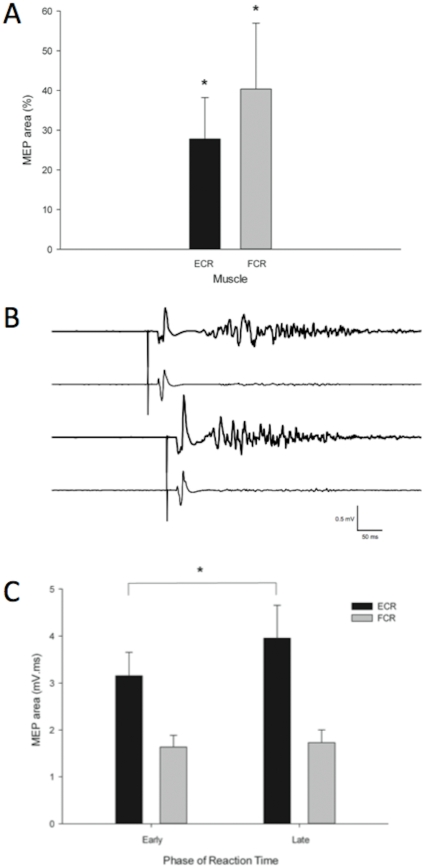
Neurophysiological and functional effects of MIR on corticomotor excitability (CME) and selective facilitation of ECR MEPs during the reaction time (RT) period preceding voluntary wrist extension (Exp 5). **A.** Each bar represents the average of 12 participants. At rest, CME was increased after MIR as indicated by a Time main effect and facilitation of MEP relative to baseline. **B.** Example EMG traces from a typical participant showing MEPs in ECR and FCR during the reaction time (RT) period of the pre-movement facilitation paradigm. Thick traces are ECR. TMS was applied early (top) or late (bottom) in the RT interval. Pre-movement facilitation is evident in ECR but not FCR, as increase in MEP size from early to late. **C.** Each bar represents the average of 12 participants. ECR and FCR MEP area during RT interval preceding wrist extension. ECR MEP area increased from early to late in the RT interval whereas FCR MEP area did not. There was no effect of Time or any interaction. * *P*<0.05, ANOVA; ** *P*<0.01, corrected one sample t-test. Error bars = 1 SE.

There was no main effect of Muscle on normalised post MEP area and no interaction between Muscle and Time (both *P*>0.4) indicating MEP area of both ECR and FCR remained stable from Post_0_ to Post_30_. Therefore post MEP areas were pooled across time points for each muscle. One-sample two-tailed t-tests indicated that ECR and FCR MEP area were facilitated after MIR (ECR 127.80±10.41%, *t*_11_ = 2.67, *P* = 0.022; FCR 140.34±16.61%, *t*_11_ = 2.43, *P* = 0.034; Figure 5a). As in Exp 1, these results indicate that MIR facilitates M1 excitability for at least 30 minutes.

#### SICI

Active-passive MIR movement had no effect on SICI in either ECR or FCR (p>0.7) There was a main effect of Muscle because FCR exhibited more inhibition than ECR (FCR 70.46±3.15%, ECR 61.12±4.89%, *F*_1,11_ = 5.65, *P* = 0.037). However there was no significant interaction between Muscle and Time indicating that %INH remained stable in both ECR and FCR throughout the experiment.

#### Reaction Time and Pre-movement Facilitation

There was no effect of Time on RT (*F*_3,33_ = 1.07, *P*>0.3), which indicates that reaction time remained stable throughout the experiment. Example EMG traces during RT/PMF task are shown in Figure 5b. For NC MEP area during RT, there was an interaction between Muscle and Phase (*F*_1,11_ = 5.60, *P* = 0.046). No interaction was found between Time and Muscle (*P*>0.7), which indicates that MEP area was not influenced by MIR active-passive movement. Therefore the data were pooled over Time for each Muscle. Paired t-tests indicated ECR MEP area increased from the early to late Phase of the RT (*t*_11_ = −2.23, *P* = 0.042; Figure 5c) as expected, while FCR MEP area did not change from Early to Late Phase (*P*>0.4).

#### Pre-trigger RMS EMG

Pre-trigger rmsEMG recorded during rest remained below 10 µV (ECR: 4.1±0.3 µV, FCR 3.8±0.7 µV). During the PMF task the baseline rmsEMG remained below 10 µV (ECR: early 5.5±0.8 µV, late: 5.8±0.9 µV, FCR: early 4.6±0.6 µV, late: 4.7±0.7 µV). There were no main effects or interactions (all *P*>0.08).

#### Active-passive movement

All participants completed 1200 cycles of MIR active-passive movement in 20 minutes. The rmsEMG in the active arm (ECR: 42±7 µV; FCR: 31±4 µV) was greater than in the passive arm (ECR: 10±2 µV; FCR: 7±1 µV) as revealed in the expected main effect of Arm (F_1,11_ = 92.26, *P*<0.0001), and no other main effects or interactions (all *P*>0.1).

## Discussion

The main finding was a sustained increase in resting passive hemisphere corticomotor excitability after 20 minutes of bimanual active-passive movement made in a mirror symmetric, but not alternating, pattern. This increase in CME was accompanied in part by pattern-dependent modulation of LICI, without accompanying changes in SAI or SICI. There was reduced IHI from the active to the passive M1 after MIR but not ALT movement, and differential IHI modulation between patterns 15 minutes post-movement. The sustained increase in ECR and FCR MEP area after mirror symmetric movement was replicated when movements were performed with a portable device. While corticomotor excitability was facilitated for both ECR and FCR, selective pre-movement facilitation was maintained prior to voluntary wrist extension. The rate of motor learning in a peg placement task was enhanced during mirror symmetric movement priming compared to two control conditions. Together, these findings indicate that mirror symmetric active-passive movement may be an effective means of facilitating corticomotor excitability and accelerating motor learning without degrading spatial selectivity of voluntary muscle activation.

### Increased corticomotor excitability after mirror symmetric movement

First, it is worth noting that there were no differences at baseline (before active-passive movement) on any metrics between the MIR and ALT sessions in Exp 1–3, and that ALT movement did not affect CME. While it is possible that ALT movement produced inhibitory and facilitatory effects that summated and cancelled along the cortico-motoneuronal pathway, this seems unlikely. ALT movement also had no effect on SAI, LICI, IHI or spinal excitability measured with H-reflex. Therefore, a null effect on CME after ALT movement seems to be the most parsimonious explanation.

At rest there was a 20–40% increase in CME for the passive ECR and FCR that was maintained for 30 minutes after mirror symmetric movement, as observed in Exp 1 and replicated in Exp 5. This sustained increase in excitability is comparable to that induced by noninvasive stimulation of M1 such as high frequency rTMS [33], [34], theta burst stimulation [35], anodal tDCS [36], and techniques which include peripheral stimulation such as paired associative stimulation [15], [17], and synchronous sensory stimulation [37]. While repetitive motor practice in the context of skill learning can increase M1 representation area [38], [39], to our knowledge this is the first demonstration of a persistent effect on M1 excitability after repetitive movement occurring in the absence of skill learning. It is worth considering how these pattern-dependent increases on M1 excitability might occur.

It seems unlikely that the facilitated ECR and FCR MEPs after MIR were due to increased alpha motoneuron excitability because there were no increases in FCR H-reflex amplitude after MIR in Exp 3, and no effects or interactions on H-reflex amplitude with Pattern. In all respects the protocol of Exp 1 and 3 were identical. Although an absence of H-reflex modulation does not conclusively rule out modulation of excitability at the spinal level, it does seem likely that the increase in CME after MIR occurred at least in part at a supraspinal level. Overall, measures of intracortical inhibition obtained from SAI, SICI, LICI and IHI as potential mediating mechanisms were inconclusive, but tended to favour a GABA_B_ receptor-mediated pathway. This is because there were no pattern effects on SICI or SAI, but some indication of reduced LICI after MIR compared to ALT (Exp 1), and reduced IHI from right M1 to left M1 after MIR but not ALT (Exp 2). Each of these is discussed in turn.

### Limited evidence of primary motor cortex disinhibition after mirror symmetric movement

In Exp 1 LICI in the passive hemisphere ECR and FCR representations was less after MIR compared with ALT movement, whereas there was no persistent change in SICI after MIR movement in Exp 5. LICI and SICI are mediated by separate neuronal populations [40]. LICI preferentially reflects activity in GABA_B_ receptor activity in M1 [40], [41]. Neurons responsible for LICI are neuromodulatory controlling a more widespread release of GABA. They inhibit neurons responsible for SICI pre-synaptically, and also directly inhibit corticospinal output neurons post-synaptically. However, within-hemisphere mechanisms of LICI, SICI or SAI do not seem capable of completely accounting for the pattern-specific effects on CME, because in isolation LICI was not reduced relative to baseline after either session, and there was no change in SICI after MIR. Therefore, a within-hemisphere modulation of intracortical inhibition alone cannot explain the increased CME after MIR observed in Exp 1 and 5.

Exp 2 provided evidence of reduced IHI from the active to the passive hemisphere after MIR but not ALT movement. However, it is difficult to directly relate the effects of MIR movement on IHI and CME. This is because IHI was measured from pre-activated (previously passive) right FCR. The voluntary activation task was chosen because it provided a stable estimation of IHI in FCR at stimulation intensities that could be adjusted precisely for maximum sensitivity [29]. Although IHI was modulated in a pattern specific manner, the time course of this modulation did not overlap perfectly with the facilitation of CME observed in Exp 1. IHI was reduced after MIR at 15 minutes, but with no difference between MIR and ALT (or from baseline) at 0 or 30 minutes (Fig. 2E). Early but not late modulation of IHI within a similar time frame has been previously reported after M1 rTMS [42]. The differences in the time course of effects on IHI and CME may have been due to the activation state, but this cannot be known for certain. Overall, IHI from the active to the passive M1 was reduced after MIR but not ALT, and reduced compared with baseline, 15 minutes after movement. This may contribute to the facilitation of CME in the passive M1 after MIR.

Interhemispheric inhibition arises via activity across the corpus callosum through dense projections between left and right M1 that serve to inhibit corticospinal output neurons. The function of these pathways is essential for unimanual movement execution and execution of independent bimanual movements [43]. These glutamatergic excitatory pathways are presumed to produce net inhibition in the contralateral M1 through their terminations onto the same GABA_B_-mediated inhibitory neurons that mediate LICI [44]–[47]. While it may have been that neurons mediating IHI and LICI have some part in enhanced CME observed in Exp 1 and 5, this issue remains inconclusive.

More definitely, it seems clear that presumed interactions between somatosensory and primary motor cortex that contribute to SAI were neither modulated after active-passive movement, nor affected by pattern. This was somewhat surprising. Previous studies have shown that primary Ia afferent pathways from muscle spindles are responsible for ECR and FCR MEP modulation during passive movement [48], [49]. As such, it was expected that any persistent increases in CME would have measureable effects through SAI mediated by somatosensory cortex. It could be that the distal cutaneous stimulation using ring electrodes, and somewhat long ISI, were suboptimal for modulating forearm corticomotoneuronal pathways, although the protocol has previously been shown to effectively produce SAI in the forearm [50]. Whatever the reason, it may be that afferent input from the periphery is not sufficient to drive persistent disinhibition of the passive M1. There is some evidence that CME is not reliably increased after passive movement alone [51]. For these reasons we suspect that interhemispheric inputs may play an important role in the increased CME obtained after MIR active-passive movement.

Coupling the activity of both hands in a mirror symmetric manner is a natural tendency when performing bimanual movements [20], [22], [52], [53]. Mirror symmetric active-passive movement provides continuous and synchronous somatosensory feedback to each M1 such that the upper limbs may become functionally coupled [23], [54]–[57]. Furthermore, because voluntary bimanual mirror symmetric movements are performed most reliably, i.e. are stable by default, inhibition may decrease within M1 and between hemispheres during [23], [58] and afterward when executed repeatedly. Our contention is that repetitive mirror symmetric bilateral movements may increase CME to facilitate activity-dependent plasticity via the same mechanisms known to induce rapid plasticity after prolonged repetitive movement in animal motor cortex [59], [60], and which occur via GABA down-regulation [61], [62]. There is indirect evidence in support of this already from studies of patients who engage in bilateral upper limb therapy.

### Functional effects of mirror symmetric movement priming

Mirror symmetric active-passive movement priming accelerated the rate of performance improvement on a skilled manual task, compared to alternating movement priming or no movement priming. Participants completed the grooved pegboard test with their nondominant hand, and learning was confirmed by faster completion times that reached a plateau and were retained when re-tested 24 hours later. The rate of learning was accelerated during trials that were interleaved with MIR movement priming. In light of the results from Exp 1–3, this accelerated learning may be related to facilitated CME and down-regulation of GABA_B_-ergic inhibition within the nondominant (passive) M1, though the design of Exp 4 precluded a direct test of these possible mechanisms. It's possible that these neurophysiological effects of MIR movement priming created a more permissive environment for use-dependent plasticity during execution of the grooved pegboard task, leading to more rapid improvements in performance, in line with previous studies showing enhanced motor learning associated with facilitated CME [63], [64]. The present findings indicate that MIR movement priming may have beneficial effects for patients undergoing motor rehabilitation after acquired brain injury such as stroke.

However, selective muscle activation is often degraded after stroke, and sustained increases in CME after MIR could further compromise the ability to selectively recruit muscles in a task-specific manner. This was examined in Exp 5, where healthy participants performed MIR with a portable APBT device designed for use by stroke patients. Afterward there was a persistent increase in the CME of the passive ECR and FCR representations, as expected. There was no persistent effect on SICI that might lead to either mirror movements or loss of muscle selectivity, problems common to patients with upper limb impairment after stroke [65]–[69]. Selective muscle activation was examined with single-pulse TMS to measure the pre-movement facilitation of MEPs in ECR and FCR during a voluntary wrist extension reaction time task, where MEP facilitation is expected in agonist but not antagonist muscles [70]–[72]. MEPs in ECR, but not FCR, increased from early to late in the RT period, both before and after MIR movement priming. This indicates that the generalized facilitation of CME produced by MIR movement priming does not degrade the muscle-specific facilitation of CME during voluntary movement. Although this result was obtained in healthy subjects and not patients, it is encouraging that there appears to be no obligatory relationship between increased excitability after MIR APBT and task-related muscle selectivity, however the potential effects of APBT in patients with upper limb weakness after stroke may warrant further attention in terms of effects on selectivity, spasticity and mirroring.

### The potential utility of active-passive movement in stroke rehabilitation

Two thirds of patients who experience stroke are left with lingering upper limb impairment with little option for recovery of function [73]. Techniques that promote cortical plasticity may offer therapeutic potential for improved recovery of motor function for patients at the chronic stage [74]. There is already some evidence that synchronous activation of upper limb muscles on either side of the body may assist upper limb recovery after stroke [14], [18], [19], [75]–[79]. However, paretic upper limb weakness can make it difficult for some patients to engage in voluntary mirror symmetric bilateral movement training [78], [80], [81]. For this reason assistive bilateral approaches have been devised [19], [82]. One such approach combines active movement of the unaffected hand with passive movement of the affected hand in a configuration, in principle, identical to the mirror symmetric pattern performed by healthy participants in the present study, by using a simple mechanical device [18], [77], [83]. When used in clinical rehabilitation studies, this has been referred to as active-passive bilateral therapy (APBT). In a recent study of patients at the chronic stage after stroke, those who engaged in daily mirror symmetric active-passive bilateral therapy (APBT) before upper limb training had significantly greater improvement in upper limb impairment scores one month post-treatment than a control group that underwent upper limb training alone [18]. Furthermore, patients who used APBT before motor practice for one month showed increased ipsilesional CME and re-established IHI between the ipsilesional and contralesional M1 when examined one month afterward, whereas patients who underwent training alone exhibited neither effect. The present results indicate that a single 20 minute session of MIR may induce persistent increases in CME in the stroke hemisphere, that carry over into the motor training period.

In conclusion, the present results indicate that in healthy participants, CME is increased for at least 30 minutes after mirror symmetric, but not alternating movement patterns. The enhanced CME is similar in extent and duration to that obtained after noninvasive M1 stimulation and may be mediated, at least in part, via modulation of interhemispheric inhibition. Furthermore, mirror symmetric movement accelerated motor learning without degrading selective muscle activation. This strengthens the assertion that mirror symmetric active-passive movement may be an effective priming modality for enhancing use-dependent plasticity within primary motor cortex.
